# Use and Design of Virtual Reality–Supported Learning Scenarios in the Vocational Qualification of Nursing Professionals: Scoping Review

**DOI:** 10.2196/53356

**Published:** 2024-07-08

**Authors:** Jenny-Victoria Steindorff, Lisa-Marie Redlich, Denny Paulicke, Patrick Jahn

**Affiliations:** 1 Health Service Research Working Group, Acute Care, Department of Internal Medicine Faculty of Medicine, University Medicine Halle (Saale) Martin-Luther-University Halle-Wittenberg Halle (Saale) Germany; 2 Interdisciplinary Center for Health Sciences Institute of Health and Nursing Science Martin-Luther-University Halle-Wittenberg Halle (Saale) Germany; 3 Department of Medical Pedagogy Akkon University of Human Sciences Berlin Germany

**Keywords:** scoping review, generalist nursing education, digital teaching formats, virtual reality application, co-creation

## Abstract

**Background:**

Numerous reviews advocate using virtual reality (VR) in educational contexts. This medium allows learners to test experiences in realistic environments. Virtually supported scenarios offer a safe and motivating way to explore, practice, and consolidate nursing skills in rare and critical nursing tasks. This is also cited as one of the reasons why VR can significantly increase the knowledge acquisition of nursing students. Nevertheless, studies are limited in their significance owing to the chosen design. Despite great interest, this results in a low level of confidence in VR as a curricular teaching method for nursing education. Therefore, defining concrete design and didactic-methodological parameters that support teachers in the use and implementation of VR is more relevant.

**Objective:**

This scoping review aims to provide an overview of significant design aspects for VR scenario conception and its transfer to generalist nursing education to generate value for the development of teaching scenarios and their sustainable implementation in teaching.

**Methods:**

A comprehensive literature search was performed using the MEDLINE (via PubMed) and CINAHL databases, and the PRISMA-ScR (Preferred Reporting Items for Systematic Reviews and Meta-Analyses Extension for Scoping Reviews) checklist was applied. The search was conducted from May to July 2022, using a specific search principle corresponding to the focus and the growing study corpus. A previously defined “population, concept, and context” scheme was employed as the basis for the double-blind review of all relevant international German and English publications released up to May 1, 2022.

**Results:**

In accordance with the predefined selection procedure, 22 publications were identified. The identified aspects aided in the development of design, didactic, and research recommendations. The intuitive operation of realistically designed VR scenarios, which are standardized, reliable, and modifiable, as well as clear instructions and specific multimodal feedback functions were described positively. The same applied to the linear structure of the sequences with graduated demands and high image quality for increased immersion with low sensory overload. Changes in perspectives, multiuser options, dialogs, and recording functions can contribute to an interactive care practice. On the research side, it is advisable to define VR terminologies. In addition to considering larger samples, varying settings, and financial issues, it is recommended to conduct long-term studies on knowledge acquisition or improved patient outcomes.

**Conclusions:**

VR scenarios offer high potential in the context of nursing education if teachers and learners develop them co-creatively according to design features and implement them by means of a well-conceived concept. VR enables trainees to develop practical skills continuously in a standardized way. In addition, its deployment supports the sensitization of trainees to digital nursing technologies and the expansion of their digital skills in a practical setting. Furthermore, it allows sustainability issues to be addressed.

## Introduction

### Background

Germany and the German health care system are facing enormous challenges. In addition to an increase in the need for care, demographic change is also leading to a blatant and increasing shortage of skilled nursing staff. Thus, the care gap in Germany is growing in all areas of care, and the need for nursing staff will rise alarmingly to 500,000 by 2035 [1]. However, in addition to the quantitative needs, the complexity of care is also increasing. A multimodal approach that considers other solutions in addition to human resources is required to counter the care crisis effectively [2]. Increasing technologization and digitalization in the health care sector can not only provide relief and additional security, but also strengthen the availability of current and person-centered (specialist) knowledge and skills in training and further education [3]. Accordingly, the educational pathways for the health care system in Germany are also changing. On the one hand, the academization of nursing education is being discussed and implemented in model study programs, while on the other hand, the new curricular orientation provides for generalist nursing education [4].

One recommendation therefore advocates transformative learning approaches that enable trainees and existing nurses to deal constructively and reflectively with the changing processes of an increasingly complex care reality.

### Virtual Reality as a Transformative Learning Approach in Nursing Education

Gradually, more educational institutions of health care are making use of virtually supported teaching-learning scenarios [4], as they represent a suitable medium to train or support the skills of health care professionals.

One of the potentials is attributed to the immersive effect [5]. With the help of virtual reality (VR), users immerse themselves in a computer-generated synthetic environment [6] and perceive it via the senses of sight and hearing and increasingly via movement and touch as well. Therefore, immersion refers to the objective degree of sensory reality fidelity. According to Milgram et al [7], the degree of reality representation, which originally referred primarily to visual representation and has since been extended to haptic or acoustic experiences, can be classified on a “virtuality continuum” between the extremes of reality and VR.

As there is no standardized usage of the term VR yet, we applied the description of immersive virtual reality simulation (iVRS) according to Shorey et al [8] as a working definition, which was decisive for determining the study inclusion and exclusion criteria as follows: “The virtual world [also “virtual reality,” authors’ note] is a 3D computer environment that provides users with interactive experiences of an alternate reality in which they are avatars who can move, sense, touch, and act upon simulated objects that appear real [9]. There are 2 variations of virtual worlds, namely, desktop virtual reality simulation (dVRS) and iVRS [10]. dVRS, also known as non-iVRS, is where users interact with an environment displayed on a computer monitor using a mouse, keyboard, touchscreen, or joystick [11]. In contrast, iVRS provides a complete simulated environment where the user is equipped with several sensory output devices such as a head-mounted device, stereoscopic unit, audio device, and haptic device [10]. It involves a higher degree of interactivity compared to dVRS — by blocking out many visual elements of the real-world environment and inducing sensory stimuli that correspond with the virtual environment, it enables the user to immerse in the virtual environment [12].”

Nevertheless, both these varieties use the principles of interaction and user participation in addition to the characteristic of graduated immersion [13]. This characteristic enables nursing trainees to experience both routines and the complexity of rare or dangerous care tasks in an activating but safe and motivating environment [14].

In the last decade, various international studies have investigated the application of VR for educational purposes in nursing. As an interesting complement to traditional teaching methods, the use of VR to improve the teaching of basic nursing skills, communication, or teamwork [15] has increased. Here, above all, the possibility of conveying abstract and complicated content is used, as one’s actions and their effects are brought into focus and the learning content is perceived as more attractive [13] and is addressed via several sensory channels in parallel [16]. Beyond this, the procedure for learning and acquiring skills and competencies, which trainees can repeat as often as needed, promotes neuronal linkage [17,18] and the resulting confidence in action. In following this approach, ways of translating theoretical knowledge into practical skills and abilities emerge [19]. However, learners and teachers have described this theory-practice transfer as critical and inadequate if only conventional teaching methods are used [13]. This can lead to not only inadequate care but also dropouts from training as trainees demonstrate an excessive demand for the learning content and its transfer to concrete practical requirements [20], especially since the number and regularity of patient contacts during training are often insufficient.

Accordingly, technology-supported teaching-learning arrangements can provide multiple services as follows:

1. They can take up the changing range of professional tasks in nursing, depict them, and teach the competencies required for this in a situational and interactive way in a safe learning setting or support the acquisition by opening up opportunities for self-observation and self-reflection [5], particularly for complex action situations that occur rather rarely in care practice and cannot be guaranteed or practiced in the training phases.
2. They can increase the intrinsic motivation to learn and the attractiveness of training [18] and can make it more effective [21].
3. They can indirectly fulfill the demand for the inclusion of digital-related competence requirements in curricula [22].

In order to establish a connection between educational and care contexts and thus provide educational value, digital technologies should be used as a learning medium in a reflected and justified manner. This makes it more relevant to define concrete design and didactic-methodological parameters that support teachers in the use and implementation of VR in their teaching.

Despite the increasing number of publications on VR as a learning medium in the educational context of health care, there is still a lack of a merger between best practice experiences and recommendations for targeted use and specific design in generalist nursing education. To our knowledge, this didactic-methodological approach to VR-supported nursing education has not been applied yet. Based on this, our scoping review is intended to contribute to showing the potentials and indications of VR as a specifically selected and supplementary teaching-learning medium and to reveal the needs of this distinctive target group for an efficient design.

### Study Objectives

The aim of this comprehensive literature review is to compile the findings and best practice examples of projects on VR-supported educational processes in nursing that have already been completed or are still in progress. With the help of this exploratory overview of the currently available evidence, it should be possible to make statements and recommendations as to which design aspects are relevant for the conception and use of didactically and methodologically significant virtually supported teaching-learning scenarios in the professional qualification of nursing specialists and to what extent these can be transferred to basic nursing training.

## Methods

### Overview

This scoping review, based on the JBI methodology [23], has obtained and mapped an overview of previous and current international research projects [24], and it is as broad and in-depth as possible [23]. With the help of the procedure described by Arksey and O'Malley [25], which comprises the steps of searching for and identifying relevant studies; selecting them; presenting the data; and compiling, summarizing, and reporting the results, it is possible to both make use of the research results already generated and identify the research gaps that still exist [25].

### Search Strategy

From May to July 2022, a comprehensive search was conducted in 2 specialist databases (MEDLINE via PubMed and CINAHL via EBSCO) according to predefined inclusion and exclusion criteria, which are presented in Textbox 1. The search strings for the literature search in the databases are presented in Multimedia Appendix 1. In addition to publications identified in the reference lists that appeared to be suitable according to the keywords and were available as full texts, grey literature from other databases and websites available online was also taken into account and included in the screening of abstracts and full texts.

**Inclusion and exclusion criteria.**

**Inclusion criteria**

***Publications***

- All publication types.
- Publications until May 5, 2022.
- Available full text (author request if applicable).
- German or English publications.
- International studies.

***Population***

- Trainees, students, and teachers in nursing care.
- Working nursing professionals participating in continuing education programs.

***Concept***

- Virtual reality (VR) applications in the professional qualification of nursing staff:
  - VR
  - Immersive applications
  - Use of a head-mounted display
- Outcome:
  - Effectiveness
  - Acceptance
  - Trust
  - Usability
  - Design features

***Context***

- Basic training as a nursing professional.
- Basic studies to become a nursing professional.
- Continuing education and training for nursing professionals.
- Interprofessional teaching-learning settings in which future nursing professionals also participate.

**Exclusion criteria**

***Publications***

- Publications after May 5, 2022.
- Full text subject to a fee.
- Non-German or non-English publications.

***Population***

- Exclusively students of human, dental, or veterinary medicine.
- Exclusively practitioners of human, dental, or veterinary medicine.
- Exclusively trainees of other health professions.
- Exclusively practitioners of other health professions.

***Concept***

- Other simulation-based teaching-learning forms without VR or immersive approaches.
- Programming aspects of VR applications only.

***Context***

- VR applications in medical-therapeutic settings without an educational purpose.
- VR applications in other educational or recreational contexts.

### Study Selection

The online tool “Rayyan” [26] was used for the consolidation and further processing of internationally published German or English articles, which were initially selected on the basis of the title and abstract. With the help of this tool, the research team was able to process the data set independently and in a blinded manner on the one hand but still cooperatively on the other. In this way, articles published up to May 1, 2022, were checked for their suitability with regard to the research question, and relevant hits were identified and extracted in a structured manner. No selection was made with regard to the study design, but both the population involved and the technologies used were taken into consideration. Therefore, we included studies in which nursing trainees, students, and teachers or nursing professionals in further education or training tested the use of VR in the form of head-mounted displays as a medium in targeted teaching-learning arrangements or helped to shape the development process. Of relevance here were, above all, statements on questions of effectiveness; information on increases in knowledge, technology acceptance, and usability; and concrete information on the didactic design of scenarios.

For this reason, publications that focused on other forms of VR representation (eg, nonimmersive 2D representation on a screen or Cardboard app–based models) or use as an assistive technology in nursing or medicine, or focused exclusively on other health professions were not considered. Furthermore, studies that focused on technological details and programming issues, but did not address the educational context, were also excluded.

### Data Extraction and Synthesis

To systematically extract, summarize, and present the information on the current state of science that is relevant to answering the research question, the included studies were first processed narratively in a data table. The analysis and structuring of the data were carried out in terms of the study characteristics and the categories deduced in advance. Accordingly, the upper categories “creative design aspects,” “methodological-didactic indications for use,” and “research recommendations” served as a tabular and thematic structural basis for the present evidence synthesis (Multimedia Appendix 2 [8,27-47]). The category “general conclusions” included further relevant statements that did not fit into these categories.

## Results

### Research and Selection of Studies

**Figure 1:**
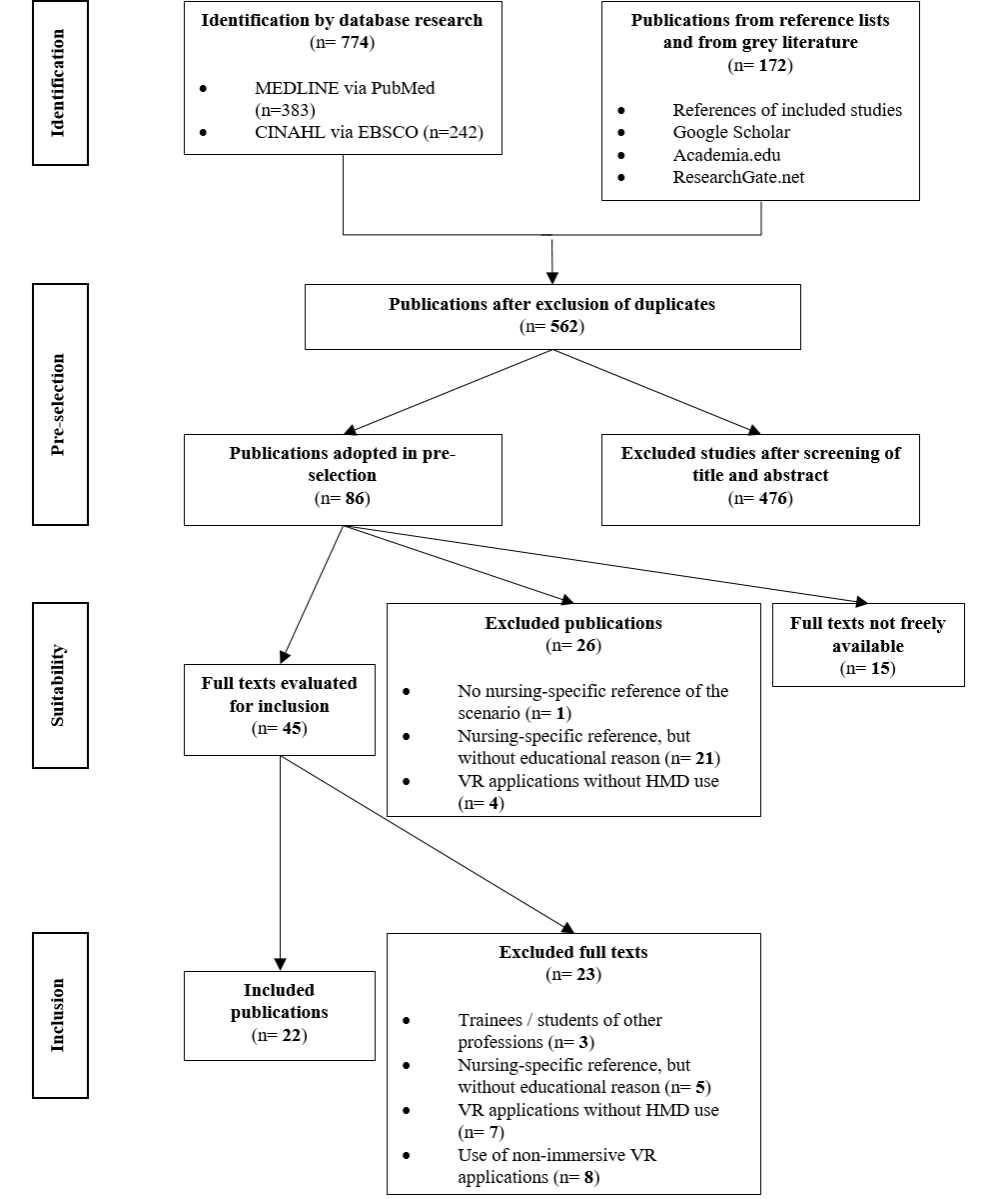
PRISMA (Preferred Reporting Items for Systematic Reviews and Meta-Analyses) flowchart for literature selection. HMD: head-mounted display; VR: virtual reality.

Through a comprehensive database search, 774 potentially relevant studies were initially found. These were supplemented by 172 publications from a hand search. Studies automatically identified as duplicates by the program were only excluded after an additional manual cross-check, and 562 studies initially remained for the review process. The preselection of 45 articles, which was carried out by a double-blinded examination of the titles and abstracts according to previously defined criteria, led to the evaluation of the full text according to the inclusion criteria. Eventually, the data synthesis included 22 articles. There were no conflicts between the independent reviewers during this process. Figure 1 depicts this procedure graphically in the form of a PRISMA (Preferred Reporting Items for Systematic Reviews and Meta-Analyses) flowchart [48].

### Characteristics of the Studies Included in the Assessment

The 22 studies included in the assessment were from 14 different countries. Of the 22 studies, 8 were from the United States [31,33,35,36,40,42,46], with 1 co-authored by researchers from Australia [39]; 2 from Ireland [27,47]; 2 from Germany [29,45]; and 1 each from Switzerland [37], Belgium [43], Scotland [28], Norway [8], Canada [43], Brazil [30], South Africa [34], Singapore [8], South Korea [38], and Taiwan [32]. The publication language was mainly English, apart from 1 study, which was published in German. The date of release of more than 77% of the studies was between 2020 and 2022, and only 5 had been published between 2014 and 2019. This is probably due to the fact that VR technologies have become significantly more affordable in recent years through several manufacturers and have thus found their way into private households as well as practical application contexts, with accompanying research. With regard to the study design, the publications were very heterogeneous as is to be expected in a scoping review. Most of the articles involved mixed methods studies [28,30,34-38,40,41,43,45-47]. Moreover, there were 2 qualitative studies [27,32], 2 experimental studies [33,41], and 3 theoretical papers [31,39,42]. Some of these studies were also partially cited in 3 included systematic reviews [8,29,44]. Therefore, this review attempted to reveal aspects that provided hints and recommendations to the chosen categories for the development of virtually supported teaching-learning scenarios for nursing trainees from among different types of publications with regard to their divergent study objectives, settings, and populations. On the basis of this, the research team scanned and divided the publications into groups according to their contribution to one or more of the 3 predefined categories. However, this scoping review aimed to provide a summary of the results of interest and not an all-encompassing presentation of the results. Table 1 illustrates the contribution of the included studies to the deductive categories.

**Table 1:** Contribution of the included studies to the deductive categories.

Study (author, year)	Creative design aspects	Methodological-didactic indications for use	Research recommendations
Weiß et al [29], 2018	Yes	Yes	Yes
Hara et al [30], 2021	Yes	Yes	Yes
Wells-Beede et al [31], 2022	Yes	Yes	No
Chang et al [32], 2020	Yes	Yes	Yes
Adhikari et al [28], 2021	Yes	Yes	No
Ma et al [33], 2021	Yes	Yes	No
Botha et al [34], 2021	Yes	Yes	No
Butt et al [35], 2018	Yes	Yes	Yes
Shah et al [36], 2021	No	Yes	Yes
Saab et al [27], 2021	Yes	Yes	Yes
Schlegel et al [37], 2019	Yes	Yes	Yes
Lee et al [38], 2020	Yes	Yes	No
Dean et al [39], 2020	Yes	Yes	No
Dorozhkin et al [40], 2017	Yes	Yes	No
Paquay et al [41], 2022	Yes	Yes	Yes
INACSL^a^ Standards Committee [42], 2021	Yes	Yes	Yes
Thompson et al [43], 2020	No	Yes	No
Plotzky et al [44], 2021	Yes	Yes	Yes
Kleven et al [45], 2014	Yes	Yes	Yes
Breitkreutz et al [46], 2021	Yes	Yes	No
Shorey et al [8], 2021	Yes	Yes	No
Hardie et al [47], 2020	Yes	Yes	Yes

^a^INACSL: International Nursing Association of Clinical and Simulation Learning.

### Characteristics of the Study Participants

Of the 22 studies, 19 described the methodical approach in the empirical surveys (for the cumulative data from the 3 theoretical papers [31,39,42], reference is made here to the respective publication). Accordingly, these included a total of 1193 participants, consisting of 14 teachers, 1018 nursing students or trainees at different stages of their studies or training, and 112 learners from other study programs. The students were studying midwifery (52 participants) [47], emergency medical services (24 participants) [41], and medicine (24 participants) [41], and were participating in interprofessional courses with nursing students in which VR was used for study purposes. A total of 12 students from other nonmedical programs completed the virtual learning program as a control group [45]. One study included 49 participants from a conference, but their professions and levels of education were not explicitly stated [40]. Nevertheless, the study was included because it tested an application that is also explicitly aimed at nurses in training and practice. The sociodemographic data, which were not given in detail in all publications, showed that the participants in the learner group were predominantly female and in an age range of 18 to 36 years but were mostly younger than 25 years. Most of the studies were conducted at a single institution, while only 4 publications presented their results from multicenter studies [30,33,41,46]. Nevertheless, almost all researchers reported that the participants had heterogeneous experiences with VR at the time of the first surveys.

### Potential of Implementing VR Into Nursing Education

The included studies depicted a variety of potentials and didactic contexts in which virtually supported teaching-learning scenarios can efficiently supplement conventional teaching methods.

Due to the almost unlimited scope for design, virtually supported teaching-learning scenarios offer a wide range of content-related practice areas for future nursing professionals. This can range from free practice and reflection of communication occasions to technology-assisted patient assessments and nursing actions [36].

The acquisition of knowledge with VR scenarios is based, on the one hand, on the theory of situated learning [8] in order to promote an active connection of didactic principles with clinical competencies [27,28,30,35,36,43,46]. The learning process in VR takes place in the context of specific action goals, competencies, structures, and rules of the simulated nursing action [49]. It also offers a way in which interactions can be experienced and practiced in the social context of a “Community of Practice (CoP)” [50]. On the other hand, VR offers a way for experiential and constructivist learning [8,27,33,37,43,47] by allowing learners to gain meaningful and realistic experiences, even in stressful, rare, and dangerous situations [28,30,37,38,40,41,47]. This comes into play especially when conventional teaching methods can only deficiently depict those situations or if it is important to control them more intensively than in the reality of care [39]. In this way, safe; low-risk; contactless; and shame-, stress-, and fear-free learning is possible [8,27,28,30,32,36,44] when the paradigm of experimental knowledge acquisition and the associated trial-and-error strategy [27,28,32] can be considered its basis. Learners thus perceive less direct pressure that can be exerted by teachers during exercise [32] and can acquire a better understanding of the relevance and effects of individual action steps through directly experienced and concrete consequences [38,46,47]. Combined with gamification elements, trainees enter into a playful learning experience with a positive culture of error [30,37], which, when used sensibly, is able to increase interest and engagement in learning as well as motivation and willingness to actively acquire and discuss the learning content [8,28,30,34,35,37,43,45-47]. While this indicates a positive added value for a better and more satisfying learning experience [27,34,38], the use of VR-supported scenarios can emphasize increased self-confidence and self-awareness from a didactic perspective [27,28,32,43]. The pride and perception of the enhanced competence of learners as well as the playfully conveyed pleasure in a challenge or a competition with fellow students can promote the attention and memorability of the content [27,28,47].

While traditional teaching methods (eg, teaching in the skills lab) require observation and subsequent assessment of performance by a teacher, VR allows learners to gather experiences and impressions unobserved but still in a kind of safe space [46] and to discuss and analyze them later with teachers. As an assistive teaching-learning tool that usefully expands and supplements existing methods [27,28,32,44], VR could replace up to 50% of the clinical hours in the conventional teaching of nursing students [36]. Thus, both novices and experts [30,44] can benefit from it during training (eg, improvement of soft skills such as empathy, interprofessional communication, and collaboration) [29,36,44,45]. Here, the function of the change of perspective or the location-independent multiplayer game option is suitable, which places learners in a realistically depicted setting of care in a targeted situation (eg, communicatively challenging situation of case discussion or family counseling) and thus extends conventional role play [44,45]. Similarly, in this environment, it is possible to expand competencies, such as observation and reflection, on a case- and action-related basis or to look at doubtful situations from different perspectives and then discuss them together with the teacher or in a class group [30,47]. Accordingly, the deliberate use of virtual scenarios in generalist nursing education is well suited to acquiring new knowledge for the first time in a multisensory way, consolidating it through repetition or different action requirements, and forming abstract concepts in an experience-based and feedback-supported way [27,28,32,46,47].

Above all, the option of applying theoretically taught content with the accompanying required practical skills and abilities of nursing in a situation-oriented manner and connected with virtual persons who require care enables learners to gather and reflect on practical experiences even before their first clinical assignments. This, in turn, can result in them being more courageous, motivated, and committed in their active engagement with real patients. On the other hand, they might also experience a feeling of greater competence and self-efficacy, which can reduce the theory-practice transfer that is often perceived as difficult [27,30,36,38,42-47]. Although the respective studies had some limitations with regard to design and generalizability, some research teams reported objectively ascertainable cognitive, procedural, and psychomotor gains, in addition to the rather subjectively assessed personal and affective added values.

However, VR is not suitable as a stand-alone teaching approach that can or should replace teaching without specific instructions and guidance [27]. Rather, it is a matter of meaningfully integrating the possibilities offered by immersive virtual simulations into the teaching of prospective nurses or into the continuing education and training of nurses who are already working.

### Didactic-Methodological Recommendations for the Use of Virtually Supported Scenarios in Nursing Education

The possible values of virtually supported teaching-learning scenarios in generalist nursing education are numerous. Nevertheless, there is a need for some didactic-methodological considerations and measures to be able to use them.

The prerequisite for this, however, is the economic, intentional, systematic, flexible, and learner-centered concept that facilitates the use of this medium, which has been adapted to the respective groups of learners, their levels of skills and knowledge, their learning experiences, and previous methods [30,32,35,37,42]. According to the deliberate practice theory, exercises should be selected on the basis of clearly defined, specific, appropriate, and measurable learning objectives that correspond to the real requirements of nursing practice [29,30,35,37,38,42]. Thus, teachers face the task of didactically reducing the available virtual possibilities by focusing on single aspects and significant content [44,47]. Due to this and several other factors, VR is not an adequate substitute for experienced instructors to teach professional nursing [27].

Saab et al [27] emphasized that the core of the nursing values of care and compassion is still human interaction. Trainees cannot acquire these exclusively through simulations. Rather, the personal and professional experience of teachers should convey these values. Additionally, they have to stimulate an empathic curiosity to generate a greater willingness to put oneself in the situation of the person receiving care and to support an accompanying in-depth understanding of the respective situation [39]. Moreover, VR cannot replace the deepening practice with the person receiving care or those involved in care for the hermeneutic case understanding of learners, in which individualized or at least partially individualized decisions about interventions in particular cases should be made with direct communicative reflection on needs and requirements. Furthermore, the abovementioned group of authors stated that there is no adequate substitute for personal and continuous feedback from the teacher for the preparation, support, and reflection of the learning situation [27]. In addition to this reflected use in general, it requires a well thought-out concept to leverage the potential of virtually supported scenarios in nursing education.

### Considerations on Implementing VR Into Teaching

The naive use of VR for self-purposes or entertainment should only be found in the leisure sector. However, in order to make a purposeful and targeted contribution to the acquisition of skills and abilities by nursing trainees, it is important to proceed in a planned and systematic manner. Thus, Dean et al [39] called for users to not become passive VR consumers but to continue to maintain a critical, analytical, and thoughtful attitude for transferability to the reality of care. This also reflects the basic attitude of caregivers. Since future nursing professionals should always adopt a critical and reflexive attitude in the course of the increasing use of technologies in nursing practice and should also be sensitized to this in their training, this applies equally to not only teachers but also learners with regard to the use of virtual scenarios.

This also presupposes that teachers organize optimal framework conditions. The International Nursing Association of Clinical and Simulation Learning (INACSL) Standards Committee [42] and Hardie et al [47] therefore recommended a detailed prebriefing for preparation and introduction to the handling of the technology. This includes concrete preinstructions [32,38,41,44], which involve the correct use of technical devices, such as the controller [30,37], and getting used to the glasses and the changing perception [30,32]. Instructions on the associated teaching material and learning content and the requirements of the scenario should also be part of the introduction. This can be done either face-to-face with tutors or instructors or via a video [28] or interactive tutorial. Good instructions and ease of use open up the potential for learners to use VR independently of teachers and thus of location [44,46], and possibly even use a multiplayer version [35,40,44]. A final debriefing in the form of feedback sessions or accompanying reflection tasks supplements meaningful usage. This can support teachers and trainees to identify learners’ current strengths and weaknesses or to analyze and discuss discrepancies between the learning experience provided and the understanding of the nursing concepts presented or even the reality of care.

Accordingly, the use of virtual scenarios is recommended especially for smaller classes [27], so that trainers can handle the organization of the set-up of the simulations as well as the assignment and creation of rotation plans [36] in a manageable and efficient way. Under certain circumstances, the information or involvement of additional teachers should be considered [36]. Moreover, it should be considered whether specifically trained instructors or fellow students should provide support for the learners, for example, to secure the environment [43]. This also becomes relevant if trainees are given the opportunity to use or borrow VR headsets for voluntary practice in their free time in a separate room [27].

This, in turn, would not only enable the self-organized learning demanded by learners and the curricula [38] but also invalidate the argument that VR isolates users and show that it promotes social interaction [44], which is a highly relevant component in nursing.

In addition to the meaningful intention to implement VR in educational contexts, design aspects are crucial elements for using the various potentials of VR.

### Overview of the Design Considerations of Virtually Supported Scenarios

With the exception of 2 studies [36,43], all included publications contained mostly experience-based hints and recommendations regarding the design of virtually supported teaching-learning scenarios for generalist nursing education.

To empower trainees to handle care situations, specific circumstances, and various settings, virtually supported scenarios should provide a realistic, plausible, and immersive learning environment [28,38,41,45,47], which should have consistent [31], clinically correct [34], and narrative story structures [47]. Due to this, multiprofessionally composed development and research groups [32,42] have to predefine concrete learning objectives [42] adapted to the current ability and knowledge level of learners [30], whereby learners come across the subjective relevance of the scenario they have experienced [51]. This forms the basis for the deduction of the most profitable specific means, details, and features. Authentic, motivating, and moderately challenging experiences should always be the goal. Thus, focusing on typical visual and auditory details relevant to the nursing process [44,46] is a major aspect. A basic prerequisite is high image and sound quality [27,34,38,41]. By observing the correct lens focus [28] and a high refresh rate, users can read text insertions [31] or recognize facial expressions and gestures [30] more easily. Moreover, this can increase the sense of immersion and perceived spatial presence within the chosen scenario [33,38,45] and prevent motion sickness [30]. Considering the cognitive load, the INACSL Standards Committee [42] recommends the selection of the type and degree of fidelity (eg, with regard to physical, conceptual, or psychological parameters). Therefore, trainees are able to draw their attention to the respective action demands [27] and challenges of different stress levels [29,37,38]. This could be supported by the targeted use of visual cues, including color markings, highlights, or animations [30,31]. The function of pausing during an exercise [31] in order to reflect on the next steps or to record the entire exercise [8] for later discussion can help to create a critical reflective attitude toward one’s performance. In addition to realistic visual and auditory details, the integration of tactile stimuli in the sense of a mixed reality experience [46] and 360° views, which enable observation of one’s performance from different angles [31], could be useful. The change of perspective [51] or modality of experience provides a basis for the reflection and discussion of actions and reactions demanded in specific situations. Since VR allows for slipping into other roles, it can promote essential nursing skills, such as empathy [52-54], which is particularly important for recreating other life perspectives and situations. The understanding of the needs and requirements of virtual people who require care on a physiological level can be supported by interactive models visualizing anatomical structures as well as regular or pathological processes [27,45,47].

Another essential recommendation for VR learning scenarios is the use of gamification elements [55]. The implementation of game-based details in the applications enables the strengthening of memory pathways [46,47], which in turn can positively influence learning outcomes [28]. This includes, for example, scores or rewards in the form of medals, congratulatory banners, or colored lights [30]. The given feedback can additionally motivate learners to perform practical nursing activities in VR [28] and support them in the development of problem-solving [27] and procedural skills [30]. Likewise, this can be supported by time limits for the execution of individual nursing actions.

Furthermore, if the application enables the collection of game-played data [41] and, for example, allows their visualization to both learners and teachers in the form of error rates [40], it can not only document but also promote intended learning outcomes, particularly when learners use this feature to analyze the learning gains according to performance [8]. Consequently, the scenarios, which rise in complexity in more challenging difficulty levels [35], should offer the possibility of the repetition of exercises [35,44] in order to achieve an increase in competence individually and in terms of one’s responsibility. If a trial-and-error strategy [27] forms the basis for this procedure, the learning process can be positively reinforced.

However, virtually supported scenarios can only unfold their potential if the handling scenarios allow. On the one hand, uncomplicated and trouble-free handling and experience of technical possibilities can increase the learners’ sense of presence in the situation [41]. On the other hand, it is an essential factor in the prevention of motion sickness [30]. Therefore, designers should pay attention to a high degree of correspondence between the image and the respective head movement, and the use of high-resolution graphics and the mitigation of technical overreactions, for example, can be useful when reaching for objects in virtual space [8,46].

Thus, design aspects should address the questions of handling and acceptance and the associated benefits for learning.

### Research Recommendations

Owing to the greater availability of and interest in the use of VR as a teaching-learning medium, the corpus of studies in the field of nursing education has grown immensely in recent years. Nevertheless, the studies included in this review have stated the inconsistent use of VR terminology, indicating the need for an unambiguous definition in publications [29,44], and have mentioned the requirement for further research with larger samples and associated statistical analysis [27,29] with regard to various aspects.

On the one hand, this involves the investigation of technical parameters and interactive possibilities, such as stereognosis [32], motion tracking, and the integration of haptic devices, enhancing VR interface elements or social media and other mobile technologies to enable collaborative learning and effective distribution of educational content [45]. On the other hand, there is a demand for further investigation of the learning process itself by means of virtual simulations and the transferability of learned nursing-relevant content to real clinical practice [30]. Shah et al [36] recommended quantitative ethnography as a possible research method to take a closer look at associated emotions; ways of thinking and acting during immersion; and how, why, or when learning groups differ in this respect. If researchers use such comparative studies, for example, to analyze several sessions with the same and different instructors and assess learners’ perceptions during the instructions in prebriefings and debriefings or with regard to different content [36], they should take care to pilot the study [42] and to provide comparable test conditions for participants in the control group so that they can, for example, walk through a real patient room in search of faulty aspects of patient and workplace safety [37]. They should ensure almost the same conditions when surveying individual learning experiences [42], learning gains [36], and long-term knowledge retention or improved patient outcomes [35]. Studies for examining and evaluating the use of VR scenarios in education in more detail should also survey possible previous VR experiences of users [41] to be able to consider possible influencing factors or risks of bias.

For the use and design of virtually supported teaching-learning scenarios for generalist nursing education, the integration of a best-practice simulation framework [47] (eg, INACSL criteria [42] and Jeffries’ Simulation Theory [32]) for the consideration of not only microdidactic but also meso- and macrodidactic influencing factors is recommended. Thus, in addition to design and application aspects, questions about financial effects or the return on investment [35] also come into focus, and interprofessional cooperation [32] should take these into account, especially for continuous modification and optimization of scenarios. Targeted needs assessment [42] and continuous learner and teacher involvement in development [30] are critical factors for the appropriate and economic development of an effective teaching-learning medium.

## Discussion

### Implications and Aspects of the Use of VR Teaching-Learning Scenarios

Within the framework of the literature research and the results presented, it must be stated that there are various ways to define VR, and it can encompass different devices, degrees of immersion, and interactions. Uniform definitions of the terms used would therefore be desirable [35,50]. Nevertheless, this medium in its various manifestations is generating successively more interest not only within the private leisure sector but also as a supplementary teaching-learning instrument in both general education and medical and nursing education contexts, as VR can meaningfully expand the number of methods with regard to various teaching-learning outcomes [36,38].

On the one hand, a virtual change of perspectives, role plays, or teamwork tasks in authentically depicted nursing scenarios could support the learning, practice, and repetition of personal and social competencies, such as empathy, heuristic case understanding, and targeted observation, which are relevant in the relational profession of nursing [2,39]. On the other hand, learners can consolidate procedural skills and abilities in virtually supported care situations by means of demonstrations, step-by-step instructions, and various feedback mechanisms [8]. In this way, they can safely apply theoretical content before, during, or even after a practical assignment in a concrete action situation and thus consolidate or assess their knowledge. This has the potential to soften limiting framework conditions and facilitate theory-practice transfer [2,36]. Teachers can benefit from the targeted use of VR in that they can give trainees learning tasks that are not bound to time and place, and these trainees are in turn more motivated and committed to partly self-directed teaching [41,50]. In addition, teachers and trainees command content illustrated more practically for appropriate discussion and reflection together [48,53].

Beyond the possibility of enhancing practical skills continuously in a standardized way, the use of VR supports trainees’ sensitization to digital nursing technologies and helps expand their digital skills in a practical setting. Even sustainability issues can be addressed in this way [38].

Nevertheless, it is important to note that almost all studies unanimously emphasized that virtually supported teaching-learning scenarios are still not an omnipotent substitute in teaching and that their use is rather critically reflected and well-considered at those points where conventional teaching methods reach their limits [40,46] to comprehensively prepare learners for the future role of a professionally acting nurse [38]. This scoping review offers an overview of the implications, considerations, and recommendations to develop and implement virtually supported scenarios reasonably and purposefully for educational demands in nursing education. An excerpt of the results is shown in Textbox 2.

This includes not handing out VR glasses to learners in an uncontrolled manner, but rather embedding the application methodically and didactically in the lessons in a meaningful way to ensure pre- and postdiscussions as well as parallel professional and technical support. Only then can the presented content effectively support individual learning [2,42,48].

VR is consequently highly recommended to complement the third location of learning, that is, the skills lab [50]. The complexity of the practice is only approximately representable owing to current restrictions, such as limitations in haptics or olfaction, which are of great relevance in the care sector, and the combination of these can lead to the high resemblance of daily nursing practice and the broad preparation of trainees [16,38]. In addition, technology in the field of VR will continue to develop in the future, and possibilities for realization may arise for those constraints. In the best case, this will happen based on the needs and requirements of respective target groups in multiprofessional teams and with co-creative participatory procedures [38]. Thus, further prospective research fields are emerging in addition to the current cost-benefit analyses, large randomized controlled studies in various teaching-learning settings, and surveys on improved patient outcomes [35,36,38,41], and these will offer further potential and provide focal points for investigation that need to be critically considered.

**Recommendations for the development and implementation of virtual reality scenarios.**

**Design recommendations**

- Realism and plausibility
- Attractive playful design with high image and sound quality
- Dialog-based narration
- Adoption of perspective
- Direct feedback and tangible consequences of action
- Hierarchical structure
- Data collection and reproduction
- Clear handling, navigation, and instructions
- Pause, repeat, and record functions
- Location-independent multiplayer option

**Didactic considerations**

- Assistive, activating, and motivating
- Multimodal, learner-centered, and experience-based teaching concept
- Specifically formulated learning goals
- Secure standardized environment
- Consolidation of theoretical, procedural, and application knowledge
- One-to-one support including feedback
- Situational testing
- Independent and flexible in terms of time, and repeatable as often as required
- Heuristically reflexive decision-making and problem-solving processes
- Self-confidence in processes, expertise, and communication skills

**Research recommendations**

- Clear definition of terminology
- Cooperative and co-creative development processes
- Larger samples and statistical analysis
- Varying settings and conditions
- Evaluation of improved patient outcomes
- Longitudinal studies on knowledge
- Cost-benefit analyses
- Inclusion of additional interactive functions
- Consideration of theoretical frameworks
- Integration of best-practice simulation frameworks (eg, International Nursing Association of Clinical and Simulation Learning criteria)

### Limitations

A methodological strength of this scoping review is the comprehensive and supplementary hand search conducted in parallel with the database search and the citation tracking to counteract the risk of excluding relevant hits. Furthermore, the research team used a tool for blinded analysis to avoid selection bias in the selection of studies as far as possible. Nevertheless, the initial decision for a sensitive search principle was changed in favor of a specific procedure, as there has been an enormous growth of extended reality (XR) applications in educational and medical contexts in recent years. Accordingly, there is a growing amount of research papers on a wide variety of focal points. However, these often only correspond to the previously defined inclusion criteria in individual points, and thus, they do not answer or inadequately answer the concrete underlying research questions for the selected target group or the corresponding application. This is also the reason for another limitation of the study. As nursing education in its generalist application in Germany is unique in a worldwide comparison, the largely international research results are only partly transferable to local framework conditions, teaching methods, and content, as well as the requirements in the initial training of future nursing professionals. Furthermore, the data protection regulations applicable in Germany should be taken into account. These can influence not only the choice of devices but also the processing and use of the generated data. Therefore, critical considerations are relevant in the reception and the attempt to generalize the results in other contexts, especially since the focus was on a selective collection of data and not on a dedicated analysis or detailed comparison of the studies with each other.

### Comparison With Prior Work

This scoping review reveals the results of selected publications according to a specific search principle. Although the aim was not to compare the studies with regard to the respective design or the reported results, the latter could be summarized under the deductively created paragraphs that address recommendations and considerations.

Compared with previous studies, which were partly considered in this study, it was possible to generate a general overview of relevant aspects that fundamentally characterize virtually supported teaching-learning scenarios in initial nursing education. On the other hand, the basis for the identification was the very specific context of nursing education in Germany. However, a large number of studies published thus far have focused on other study populations from the medical and general education sectors or other definitions of VR in their surveys and explanations.

The inclusion and exclusion criteria used served primarily to provide those actors involved in German nursing education and training with information on the use and development of virtually supported teaching-learning scenarios, which corresponded to the international consensus and met the needs of the German context. Consequently, this publication can serve as a point of reference for both national and international recipients, provided that they critically evaluate it and, if necessary, supplement relevant aspects, which are related to the respective country, action, teaching, or study population background.

### Conclusions

Flexible use, a positive error culture, and learning that can be individually controlled and adapted to the knowledge levels of trainees by means of virtually supported teaching-learning scenarios can increase learning motivation and satisfaction. Simultaneously and compared to other common teaching methods, VR can reduce time, personnel, and material resources, and future nursing professionals can specifically train, deepen, and consolidate the procedural, personal, and social competencies of professional nursing knowledge and actions in both theoretical and practical teaching sessions.

Nevertheless, VR cannot and should not replace experienced nursing teachers, especially to convey elementary nursing values such as care and compassion. Therefore, learners and teachers should be actively involved in the co-creative design and evaluation process of virtually supported teaching-learning scenarios for the acquisition of skills and competencies in a practical yet safe setting. This will help to reveal the needs of the target group from the beginning and to incorporate them directly into the development on an iterative basis. In addition, future users can identify weak points or errors in content or applications more quickly than nonspecialist developers who may focus on different aspects. This could also launch the systematic implementation of this medium in the curriculum. Moreover, trainees and teachers will be sensitized to apply it critically and reflectively owing to the deeper insights that accompany the process.

## Appendix

Multimedia Appendix 1 Search strings for the literature search in 2 separate databases.

Multimedia Appendix 2 Overview of the essential characteristics of the studies included in this scoping review.

Multimedia Appendix 3 PRISMA-ScR (Preferred Reporting Items for Systematic Reviews and Meta-Analyses Extension for Scoping Reviews) checklist.

## Abbreviations

dVRS: desktop virtual reality simulation
INACSL: International Nursing Association of Clinical and Simulation Learning
iVRS: immersive virtual reality simulation
VR: virtual reality
