# Supplementary material for: Use and Design of Virtual Reality–Supported Learning Scenarios in the Vocational Qualification of Nursing Professionals: Scoping Review
Source: JMIR Serious Games. 2024 Jul 8;12:e53356. doi: 10.2196/53356 (PMC11263887; doi:10.2196/53356)
Supplement: Multimedia Appendix 1 [file games_v12i1e53356_app1.docx]

**Multimedia Appendix 1.** Search strings for the literature search in 2 separate databases.

| **PubMed via MEDLINE** | ((((“nurs*”[Title/Abstract]) OR (“train*”[Title/Abstract])) OR (“student*”[Title/Abstract]))) AND (“teach*”[Title/Abstract]) AND (((“VR*”[Title/Abstract]) OR (“virtual*”[Title/Abstract])) OR (“real*”[Title/Abstract]))) AND (((“profession*”[Title/Abstract]) OR (“train*”[Title/Abstract])) OR (“qualif*”[Title/Abstract])))  OR  (“virtual reality”[MeSH Terms] OR “virtual reality”[Text Word]) AND “nurs*”[All Fields] OR “train*”[All Fields] OR “student*”[All Fields]) AND (“nurs*”[All Fields] OR “teach*”[All Fields]) AND (“vr”[All Fields] OR “virtual*”[All Fields] OR “real*”[All Fields]  OR  (“virtual reality”[MeSH Terms] OR “virtual reality”[Text Word])) AND (“profession*”[All Fields] OR “train*”[All Fields] OR (“profession*”[All Fields] OR “qualif*”[All Fields]))" |
| --- | --- |
| **Cinahl via EBSCO** | (("nurs*" OR "care") AND "educat*" AND ("vr" OR "virtual*" OR "real*" OR ("ar" OR "augment*" OR "real*") OR ("xr" OR "extend*" OR "real*") OR ("mr" OR "mix" OR "real*")) AND "immers*") |
