# Supplementary material for: Use and Design of Virtual Reality–Supported Learning Scenarios in the Vocational Qualification of Nursing Professionals: Scoping Review
Source: JMIR Serious Games. 2024 Jul 8;12:e53356. doi: 10.2196/53356 (PMC11263887; doi:10.2196/53356)
Supplement: Multimedia Appendix 2 [file games_v12i1e53356_app2.docx]

**Multimedia Appendix 2.** Overview of the essential characteristics of the studies included in this scoping review.

| **Topics** | **Author-ship and Year of Publication** | **Country of Origin** | **Study Size and Popu-lation** | **Study Design** | **Study Objective(s)** | **Results** |
| --- | --- | --- | --- | --- | --- | --- |
| Design  Didactics  Research | **Weiß S, Bongartz H, Boll S (2018) [29]** | Germany | educa-tional contexts in nursing | systematic review | survey of the current state of immersive VR with HMD in nursing education and its impact on learning | **Design**   - multisensory feedback increases the sense of presence, different environments increase the stress level and realism - significantly higher immersion in room-scale VR - good understanding of non-linguistic signs - fun despite technical deficits and nausea   **Didactics**   - VR (HMD) can support learning and increase learner motivation - better understanding of individual topics - VR is more usable and associated with more fun - can contribute to significantly higher exercise frequency and duration - no follow-up effect compared to conventional training   **Research**   - definition of the terminology of VR - still small number of studies - larger samples with statistical analysis |
| Design  Didactics  Research | **Hara CYN et al (2021) [30]** | Brazil | 13 teachers and  30 students of nursing | quantitative study design (Heuristic Evaluation for Digital Educational Games) | description of the design and heuristic evaluation of a VR application with regard to the promotion of communi-cative competence | **Design**   - involvement of the target group - Heuristics by Nielsen (1994) to ensure motivation, pedagogical quality and technical effectiveness - high quality through the selection of appropriate technology and software - increased feedback - progress recognizable through color-coded subtitles - facial expressions of those being cared for more expressive - AI integration with pattern recognition for conversational skills   **Didactics**   - learning objectives to be clearly formulated - improve initial information - users should be guided to avoid sudden movements (dizziness) - allow adaptation time to VR goggles (not to be used by spectacle wearers)   **Research**   - further studies on the transfer of the contents into practice   **General Conclusions**   - learners at all levels of education can benefit from its use - the interface increases and facilitates quality as well as immersion - some players had difficulties in recognizing, diagnosing and correcting their own communicative errors - conversational skills and attractiveness of pedagogical tools increase motivation |
| Design  Didactics | **Wells-Beede E et al. (2022) [31]** | USA | students of nursing (without quantita-tive informa-tion) | Beta testing with nursing students after initial design of the application | presentation of the application developed | **Design**   - possibility of changing the perspective - higher resolution of the text overlays   **Didactics**   - innovative and unique learning experience   **General Conclusions**   - occasional physical discomfort when wearing the VR headset |
| Design  Didactics  Research | **Chang YM, Lai CL (2020) [32]** | Taiwan | 60 students of nursing | compara-tive qualitative research design | disclosure and under-standing of nursing students' experiences of using a VR application for the purpose of the skill acquisition process    base for reflection for teachers regarding conventional and technology-integrating teaching methods | **Design**   - high responsiveness - checklists, step-by-step guidance and error alerts   **Didactics**   - use as a supportive tool for self-organized learning - increases learning flexibility, can be controlled independently - need for familiarization and concrete advance instruction on VR use - easy learning of gestures - increased interest in learning - demonstrations promote overall understanding, even if individual steps are not yet mastered   **Research**   - inclusion of stereognosis and interactive functions     **General Conclusions**   - stress-free learning environment - resource-saving (time, staff, materials) and repeatable learning - traditional teaching methods are more realistic due to the sense of touch and patient reactions |
| Design  Didactics | **Adhikari R et al (2021) [28]** | Scotland | 19 students of nursing | two-stage, sequential feasibility study in mixed-methods design | investigating the impact of VR use on the self-efficacy of nurses in training and their perception of its acceptance and applicability as a complemen-tary teaching method | **Design**   - realistic, interactive and immersive - immediate feedback - clear options - repeatability - take lens focus into account   **Didactics**   - as a complementary and supportive teaching method - safe exercise platform - significant increase in self-confidence with significant decrease in anxiety and easier acquisition of knowledge - practice of rare situations before practical experience - learning from mistakes - increased decision-making ability and self-confidence - activates individual class participation   **General Conclusions**   - easy to use, but partly twisting of the body and neck as well as dry eyes - lack of crucial human factors such as verbal and physical interaction with the team - fear of using new technologies |
| Design  Didactics | **Ma Z, Huang K-T, Yao L (2021) [33]** | USA | 69 students of nursing | experi-mental study with 2x2 between-subjects design and randomized assignment | investigating the feasibility and effectiveness of computer role-playing games with regard to nursing students' empathy  -> focus on immersion and perspective | **Design**   - immersion level and perspective have a significant effect on empathy, attentional localization, spatial situation model and perceived self-location   **Didactics**   - use of VR to convey empathy   **General Conclusions**   - no significant results regarding perceived opportunities for action |
| Design  Didactics | **Botha BS, de Wet L, Botma, Y (2021) [34]** | South Africa | 34 students of nursing (con-venience sam-pling) | experi-mental study incl. observation and question-naire survey | expansion of previous findings on the use of VR in the teaching of health professions  -> focus on experience in the application (e.g. immersion, presence, usability, degree of recommend-dation, familiariza-tion time, feasibility) | **Design**   - easy navigation and operability within the scenario - balanced picture and sound quality - consideration of technical bugs - clinical correctness and attractiveness of the virtual environment - availability of relevant information   **Didactics**   - good to use and provides a positive learning experience that is sometimes preferred over other forms of simulation   **General Conclusions**   - high overall satisfaction and recommendation of the application - SUS over 70% |
| Design  Didactics  Research | **Butt AL, Kardong-Edgren S, Ellertson A (2018) [35]** | USA | 20 students of nursing (con-venience sam-pling) | mixed-methods pilot study (conver-gence model of triangula-tion) with control group after the first develop-mental iteration + follow-up measure-ment (after 2 weeks) | usability and reaction to a VR scenario, for practicing catheteri-zation  +  comparison with traditional methods | **Design**   - coherent and immediate feedback - location-independent multiplayer option     **Didactics**   - VR application as an opportunity for independent practice: longer practice duration as well as higher number of correctly performed procedures than with conventional teaching methods - follow-up results were identical - catheterization would be practiced with the VR application rather than with the task trainer   **Research**   - need for multi-center longitudinal studies with large sample sizes - investigation of return on investment, long-term knowledge retention and improved patient outcomes   **General Conclusions**   - positive usability rating - highly activating and motivating - desire for regular use |
| Didactics  Research | **Shah M, Siebert-Evenstone A, Eagan B (2021) [36]** | USA | one teacher and 29 students of nursing | epistemic network analysis (ENA) as a method of quantitative ethno-graphy (QE) | How can Simulation Learning System with Virtual Reality (SLS with VR) be used to enhance clinical judgement and quality and safety education of nurses?  -> expanding research on immersive learning methods using theory-based learning analysis techniques | **Didactics**   - merging theoretical concepts in application with people in need of care - effort required to organize the simulations - high coordination requirement with regard to implementation and feedback on the part of the teachers   **Research**   - ENA as useful method in research of usability, efficacy und usefulness of VR |
| Design  Didactics  Research | **Saab MM et al (2021) [27]** | Ireland | 26 students of nursing (selec-tive snowball sam-pling) | qualitative descriptive study design | investigating student´s perspective on the integration of VR in nursing education | **Design**   - visualization of the body and physiological processes - adopting the patient's perspective   **Didactics**   - valuable for different types of learners and offers more individualized 1:1 teaching - VR can complement current teaching-learning methods, supports learner confidence and provides a safe space for nursing students to practice different skills and learn about human anatomy, physiology, problem-solving processes, trial-and-error strategies and clinical decision-making - recommendation for use before and between learning units and practical assignments (refresher) or after class to test knowledge - need for continuous feedback to prepare, support and debrief learners - recommendation for use in smaller classes   **Research**   - limited transferability of the results -> experimental studies with larger samples would be necessary   **General Conclusions**   - VR offers a new, easy-to-remember, inclusive and activating way of learning - challenges and dangers of implementation: - costs for purchase and maintenance as well as resources to convert texts and PPT into VR - VR can promote empathy, but is also perceived as anti-social and isolating, negatively impacting human interactions at the core of care values of caring and compassion - partly visual problems, dizziness, motion sickness and risk of injury |
| Design  Didactics  Research | **Schlegel C, Weber U (2019) [37]** | Switzer-land | level 1: 53 students of nursing  level 2: 78 students of nursing | explorative two-stage mixed-methods-design  -> Level 1: “One-Shot study-Design”  -> Level 2: "The Static-Group Comparison Design" | How do students adjust to the VR game and what impact does it have on the development of competences? | **Design**   - manual handling and navigation in VR must be simple - appropriate level of task - limitation of game duration to 5 minutes to prevent motion sickness   **Didactics**   - detailed instruction on how to use the controllers and sufficient practice opportunities - coordinated use with regard to other teaching methods - significantly better performance of the intervention group in OSCE examinations   **Research**   - study designs with larger samples to calculate power - participants in the control group should be given the opportunity to gain similar learning experiences in the practice setting   **General Conclusions**   - no or only little stress during the training with the VR goggles - high fun factor |
| Design  Didactics | **Lee Y, Kim SK, Eom M-R (2020) [38]** | South Korea | 60 students of nursing (con-venience sam-pling) | mixed-methods-study | testing of a VR simulation (mental health and schizo-phrenia) with regard to usability and usefulness as well as identification of potential for improvement | **Design**   - high degree of realism leads to higher sense of presence - high picture and sound quality - maximum duration of 10 to 15 minutes (to prevent dizziness) - consequences of actions should be visible: feedback and presentation of correct answers with explanations - (feedback) strategies that encourage repeated learning, such as game elements and individual scores - embedding of communicative sequences with the virtual carers, possibly including other devices (also for recording)   **Didactics**   - detailed introduction - better understanding of the clinical picture, possibility and courage to actively engage with the patient in a safe environment - increases the participants' commitment and motivation to learn about psychiatric nursing - situations rarely experienced and observed   **General Conclusions**   - good applicability - beneficial and exciting - builds an emotional connection with the virtual carers - helpful for future work in the clinical setting - HMD was sometimes uncomfortable to wear - effective alternative to clinical training in psychiatric care |
| Design  Didactics | **Dean S et al (2020) [39]** | Australia and USA |  | theoretical paper | questions:  Does VR reinforce a type of person to be cared for and neglect uniqueness?  Can empathy be generated?  Does VR lead to a greater distance between the person being cared for and carer? | **Design**   - involvement of teachers, users and persons being cared for in the development process   **Didactics**   - use of VR is valuable to teach procedures, but does not replace the opportunity to learn from experienced mentors how to provide care during these procedures to the person being cared for   **General Conclusions**   - VR enables sensory immersion, but is not contextual - the relationship should remain in focus (if necessary, guidelines for the use of VR in teaching) - recommendation to consider empathic curiosity |
| Design  Didactics | **Dorozhkin D et al (2017) [40]** | USA | 49 congress guests (sur-geons, anaes-thetists, nursing staff) | mixed-methods-study | investigating the usefulness, design and validity of the developed VR application | **Design**   - combination of realistic visualization and highly immersive interactive environment - high-quality feedback, e.g. determination of the error rate, in order to be able to compare the previously self-reported knowledge with the results - multi-user option for joint virtual interaction   **Didactics**   - suitable method to simulate dangerous situations (fire in an operating theatre) and to practice the corresponding behavior   **General Conclusions**   - 67% of the participants would prefer the use of conventional teaching methods (particularly high values regarding usefulness and effectiveness) |
| Design  Didactics  Research | **Paquay M et al (2022) [41]** | Belgium | con-venience sam-pling with 83 students (rescue: 24, nursing: 35, medi-cine: 24) | Experi-mental cross-sectional study (in-depth follow-up study to Servotte et al 2020) | investigating the factors influencing the users' sense of presence (SoP) and immersion experience    verification and extension of the theoretical framework according to Servotte et al (2020)  identification of key trends in order to tailor the scenarios to the respective professional group | **Design**   - consideration of the specific needs of individual professional groups when designing VR scenarios -> impact on the SoP and learning outcomes - best practice experiences should be incorporated into the design   **Didactics**   - consideration of previous VR experience (low knowledge may lead to lower SoP)   **Research**   - a clear definition of SoP is needed - further research into cognitive processes based on prior experience and memory   **General Conclusions**   - a higher SoP leads to better training performance - factors influencing the SoP: - level of previous experience - gender (women have higher SoP levels than men) - professional group affiliation (nursing students have a higher SoP level than other professional groups) - but no weakening influence by internal factors such as coping and decision making |
| Design  Didactics  Research | **INACSL Standards Committee (2021) [42]** | USA |  | theoretical paper | theoretical summary of best practice recommend-dations for the development of VR scenarios | **Design**  orientation towards the following criteria that support effectiveness:  1) multi-professional development (with experts from education, pedagogy and practice) taking into account ethical issues  2) needs assessment (possible concerns, SWOT for the institution, stakeholder interviews, definition of outcomes)  3) measurable target formulations based on the basic knowledge of the users  4) the format corresponds to the objectives  5) scenarios should provide a context for the experience  6) use of different credibility types to support realism  7) planning a learner-centered facilitative approach based on learning objectives, (prior) knowledge and level of experience and expected outcomes  8) prebriefing plan  9) debriefing or feedback session and/or guided reflection task after application  **Didactics**   - all simulation-based experiences require intentional and systematic, but also flexible planning   **Research**   - evaluation of the learners and the VR experience - piloting of the application before full implementation |
| Didactics | **Thompson DS, Thompson AP, Mc Connell K (2020) [43]** | Canada | 205 students of nursing | descriptive cross-sectional study in mixed-methods design | investigating the engagement and subjective experiences of nursing students with regard to a VR application | **Didactics**   - higher engagement of the participants when using VR in contrast to conventional (both active and passive) teaching methods - good opportunity to link and complement the content from lecture and lab - support for learning: VR helped with health assessment and consolidation of knowledge through easy use and interactive design of anatomical and physiological concepts (3D images and videos) - more control over learning content and intensity through VR - (technical) support by instructors and/or partners (securing the environment) important   **General Conclusions**   - desire for further applications in as well as outside the classroom - the Exploratory Learning Model is helpful in integrating VR into teaching |
| Design  Didactics  Research | **Plotzky C et al (2021) [44]** | Germany |  | systematic mapping review with highly sensitive approach | article overview on educational VR simulations in nursing + analysis of didactic and technical approaches | **Design**   - inclusion of haptic devices - demonstration of processes - field of view tracking - text information and text-based dialogues - change of perspective - step-by-step guidance - complementing existing technologies (e.g. manikin + vital signs) - multiplayer role-playing   **Didactics**   - didactic reduction by focusing on individual aspects - expansion of role plays and team training - improvement of procedural technical knowledge - teaching practical skills with embedded theory to reduce the theory-practice gap - training of emergency situations - repeatable and location-independent - training of soft skills such as empathy and communication - training of psychomotor skills   **Research**   - great differences in the use and definition of VR for educational purposes -> use of clear terminology |
| Design  Didactics  Research | **Kleven NF et al (2014) [45]** | Norway | 12 post-gradu-ated students of nursing + 12 students of other non-medical degree courses | two exploratory studies | investigating an educational 3D role-playing game as a training method for communi-cation, teamwork and other practical skills of surgical nurses  +  investigating the potential of virtual hospital tours for non-medical professionals+ recommend-dations on the design and development of a virtual university hospital as a place for educational activities | **Design**   - entrance tutorial - visual cues and guidance to simplify navigation in the room - 3D anatomy models - simulated health care staff and care recipients - representation of typical nursing and interactive equipment as well as flowcharts - realistic and representative scenarios for comparable situations in real practice   **Didactics**   - training of procedures and simulated patient contacts - anatomy teaching - increased engagement and motivation   **Research**   - further need for studies on immersive HMD applications - demand for clear terminology   **General Conclusions**   - sometimes difficulties in reading information from clothing, facial expressions and body language (audio seems to be the most important source) - high sense of presence and immersion, but also discomfort when wearing the HMD (motion sickness with constant movement or tension in the jaw, dry eyes etc.) - there are still difficulties in interacting with children, other cultural backgrounds or in calming down before an operation - only partly better understanding of patients (due to limited interpretation of non-verbal information) |
| Design  Didactics | **Breit-kreutz K et al (2021) [46]** | USA | 300 students of nursing | exploratory, multicenter mixed-methods utility study | investigating the user-friendliness and the reaction of nursing students to the developed VR intervention as a technology and as a learning modality | **Design**   - visual cues and feedback - realism through hospital-typical background noises   **Didactics**   - motivates engaged learning - independent, repetitive and low-material practice - better understanding and recall of individual steps   **General Conclusions**   - the assessment of usability correlates with gender (higher results for men), but not with year of study, grade point average or age (participants were, however, only between 20 and 25 years old) - however, the usability values also show that further improvements are still indicated |
| Design  Didactics | **Shorey S, Ng ED (2021) [8]** | Singapore |  | systematic review of RCTs und quasi-experi-mental studies | survey and confirmation of current evidence on the use of virtual worlds as a teaching-learning method in the form of a narrative summary | **Design**   - specific instructions and feedback   **Didactics**   - use as an alternative or supplementary teaching method for theoretical knowledge - observation and execution of error-free processes - additional practice possibilities independent of time and place - increased motivation to learn and interest in content   **General Conclusions**   - conflicting results on skill and knowledge acquisition - virtual worlds are most effective in improving cognitive learning outcomes compared to clinical skills and affective learning outcomes - no significant differences in anxiety and self-confidence - higher time-cost efficiency than simulations with manikins and face-to-face teaching - high satisfaction of participants - partly still low realism or different feeling than in reality |
| Design  Didactics  Research | **Hardie P et al (2020) [47]** | Ireland | 42 students of pediatric nursing and 52 students of mid-wifery | evaluative study with a multimodal mixed-methods approach, comprising a cross-sectional as well as an observational study | investigating the subjective experience of an iVR storytelling application with regard to the level of immersion, interaction, imagination and motivation | **Design**   - authentic reproduction of care situations with narrative elements - sequenced content - use of colors and animations - possibility to interact with anatomical objects   **Didactics**   - new interactive, emotional and sensory experiences or experiences not possible in real life - deepened understanding and improved memorization of (care) concepts - retention of attention - motivation for subsequent discussions   **Research**   - multi-center studies with larger samples increase the transferability of results - implementation of best practice simulation frameworks (e.g. INACSL) is recommended   **General Conclusions**   - positive values for engagement and fun - highest values for motivation due to significant experiences compared to traditional teaching methods - shielding from unwanted distractions and thus better focus and concentration - high sense of immersion - activation of the imagination, which in turn improves understanding and learning - contribution to problem-solving ability, however, is evaluated neutrally or negatively - uncomfortable, claustrophobic feeling when wearing the HMD - motion sickness, dizziness and neck stiffness from turning the head - distraction due to background noise if no headphones were worn - partly technical difficulties with the smartphone, the software or poor image quality |
